# Legal Notes for Institutional Workers

**Published:** 1912-08-24

**Authors:** 


### LEGAL NOTES FOR INSTITUTIONAL WORKERS

**(The Editor will be pleased to answer Legal queries in this column.)**

### Libels on Hospitals.

How often do those who write for the papers of a certain class make serious aspersions upon hospitals in general or some hospital in particular! One reads, "This unfortunate person was turned out of hospital to die in the street." Can no proceedings be instituted to prevent a vile insinuation of this character? It seems that there is no remedy by action at law. In this regard the hospital must be looked at, not as an individual having a character to protect, but as a corporation or body of persons outside the protection of the law of libel. "The words complained of," said a distinguished judge, "in order to entitle a corporation or company to sue for libel or slander, must injuriously affect the corporation or company as distinct from the individuals who compose it. A corporation or company could not sue in respect of a charge of murder, or incest, or adultery, because it could not commit these crimes. Nor could it sue in respect of a charge of corruption or of an assault, because a corporation cannot be guilty of corruption or of an assault, although the individuals composing it may be. The words complained of must attack the corporation or company in the method of conducting its affairs, must accuse it of fraud or mismanagement, or must attack its financial position." The law evidently contemplates that a corporation, if capable of suing for libel, must be a trading corporation. A hospital may lose patients if it is held up to reproach; but is that necessarily a matter for regret? Is the institution thereby damaged? These would be questions for a jury to determine.

### Criminal Liability.

Apart, however, from the civil law, the protection of a court of criminal jurisdiction might be invoked by those connected with a hospital. A libel is a criminal offence, and will be punished as such if the tribunal be satisfied that it was calculated to lead to a breach of the peace. Where aspersions are cast upon the management of a great institution or its treatment of patients a breach of the peace is by no means a remote contingency. Students have been known to take the law into their own hands before now. Nor would the fact that the allegations were true afford a defence. The defendant would have to go further and satisfy the jury that it was in the public interest that they were published to the world. The institutional manager would do well to remember that this remedy in the criminal courts is available, although an action for damages for libel would be perfectly futile.

### Hospitals and the Law of Nuisance.

The recent somewhat drastic action of the inhabitants of Lucan, near Dublin, who signified their objection to a sanatorium by pulling it down *vi et armis*, reminds us that the hospital is not always a welcome neighbour to the inhabitants of a district. In what circumstances can those who own or manage a hospital be sued for nuisance? Noise, smell, and the danger of infection may all be nuisances within the eye of the law. Neighbours are not likely to be troubled by noise in the wards, or by disagreeable odours owing to the proximity of a hospital. Moreover, something will depend upon the site of the hospital.

### Neighbourhood of the Hospital to be Considered.

It is not difficult to ascertain the law on the subject, but it is difficult to apply the law to the facts of a particular case. The question whether there is a legal nuisance from noise, etc., largely depends upon the character of the neighbourhood. As Lord Halsbury once said: "A dweller in towns cannot expect to have as pure air, as free from smoke, smell, and noise, as if he lived in the country and distant from other dwellings; and yet an excess of smoke, smell, and noise may give a cause of action; but in each of such cases it becomes a question of degree, and the question is in each case whether it amounts to a nuisance which will give a right of action."

### Nuisance by Danger of Infection.

In one respect the hospital, and more especially the fever hospital, is exposed to actions for nuisance from which other bodies generally escape. People are not unnaturally afraid of the infection which is supposed to hang like a pall over and upon the walls of a hospital for infectious diseases.

### Miner's Nystagmus.

In a case at Newcastle (July 18) an important point arose as to the disease of nystagmus. It appeared that the applicant, a miner, began to experience trouble with his eyes in February 1910, and in the following June he got a certificate to the effect that he was suffering from nystagmus. From the beginning of July 1910 until February 11, 1911, he received compensation at the rate of £1 per week. There was no further certificate from the certifying surgeon, and the employers cut down the compensation to 10s. 3d. The grounds on which the respondents denied their liability were that the certifying surgeon did not certify that the applicant was suffering from nystagmus, and therefore he was not prevented from earning wages at employment. It was argued on behalf of the employers that Parliament thought it should be made an industrial disease, and the man compensated for it while he was suffering, but it never meant that, after recovery, the employer should go on giving compensation on account of the disease. His Honour decided that, on the evidence, there must be an award for the applicant. It seemed to him to be admitted that a man who had suffered from nystagmus was more liable to suffer from it again, and he found that was the fact in this case. He made an award for the plaintiff. Costs were granted, with one qualifying fee.

